# Supplementary material for: When case reporting becomes untenable: Can sewer networks tell us where COVID-19 transmission occurs?
Source: Water Res. Author manuscript; Available in PMC 2022 Dec 20. (PMC9763902; doi:10.1016/j.watres.2022.119516)
Supplement: MMC1 [file NIHMS1859713-supplement-MMC1.pdf]

Supplementary Materials

A Sampling Design and Sample Collection

A.1 *Site Identification*

Wastewater Treatment Facilities (WWTFs): The City of Atlanta Department of Watershed Management (DWM) routinely collects samples from influent lines of WWTFs for industrial pre-treatment wastewater monitoring. With the consultation of operators at WWTFs, partners at the DWM identified sampling access points for three main influent lines entering each WWTF.

Community Sites: After manholes of community sites were identified using information on the sewer network, the manholes were screened for accessibility in the field. The manholes needed to be accessible and not located in an area with busy traffic (e.g., busy roads) or far from the road (e.g., in the woods). For manholes in grassy or wooded areas, risks were considered for trip hazards (e.g., rocks, sticks, and uneven footing), wildlife (e.g., ticks and snakes), and strenuous hikes. When a manhole was not accessible, the enumerator traced upstream to find an alternative manhole that was accessible.

A.2 *Sampling Requirements*

Permission: Wastewater sample collection was conducted cooperatively by the Emory University sampling team and the DWM team. The DWM has permission to access and sample from manholes within their jurisdiction (i.e., within the city of Atlanta). For the Emory team to help with the sample collection process, they were required to obtain City of Atlanta contractor badges which were worn in the field. The contractor badges also granted access to the WWTFs without the need for an escort from the DWM team.

Equipment: Depending on the type of samples (grab vs. Moore swab) that were collected, different types of equipment were brought into the field. For collecting grab samples, 1 L autoclavable bottles, metal bucket (with handle) and rope, and long-handled water sample dipper or a painter's pole rigged with a "seat" for the 1 L bottle to sit in were used. For collecting a Moore swab sample, fishing lines (weighted for 50-lb), cotton gauze, bendable metal (e.g., metal coat hanger), a magnetic hook, thin ropes, and a collection bag (e.g., quart-size Ziploc bag, WhirlPak, Biohazard Specimen Transport Bag, etc.) were used. In addition, tape (for labeling collection bottles), a permanent marker, a cooler, ice or ice pack(s), and personal protective equipment (e.g., disposable gowns and gloves, N-95 mask, and face shield etc.)

were used for sample collection as needed. A protocol that describes the materials and methods used in the field to collect Moore swabs and grab samples for wastewater sample collection was published on protocols.io (http:/dx.doi.org/10.17504/protocols.io.b2rzqd76).

Travel Time: For Atlanta COVID-19 wastewater surveillance, travel time between sites typically ranged from 10 to 30 minutes, cumulatively 1–3 hours of driving per day, which includes the travel time between the laboratory at Emory campus and the sampling sites.

A.3 *Sampling Schedule*

WWTFs: The DWM team collected influent line samples from Utoy Creek Water Reclamation Center and South River Water Reclamation Center. These two plants primarily serve low-income populations in Fulton County. The Emory sampling team collected samples at influent lines of R.M. Clayton plant, which primary serves high-income populations in Fulton County. Grab samples were collected from these nine influent lines every Monday morning, stored in a cooler on ice, and delivered to the laboratory on Emory campus by noon.

Community Sites: The DWM team collected Moore swab samples from manholes located within multiple low-income neighborhoods in South Atlanta. Moore swabs were placed in the wastewater stream of ten manholes on Monday mornings, retrieved on Tuesday mornings, and delivered to the laboratory by 1 PM on Tuesday. Beginning in November 2021, the DWM team began collecting Moore swabs at six additional sites over the weekend (placed on Saturday mornings and retrieved on Sunday mornings). After swabs were retrieved on Sunday, they were stored overnight in a refrigerator at 4 °C, picked up on Monday mornings at 7:30 AM, and delivered to the laboratory by 8:00 AM.

A.4 *Mobile Data Collection*

The DWM GIS team utilized Esri's Field Operations Apps, including ArcGIS Workforce, ArcGIS Field Map, and ArcGIS Survey123, to conduct the field data collection. ArcGIS Workforce allowed sampling teams to be assigned specific manholes to collect samples from thus avoiding confusion if multiple manholes were present in the area. A dispatcher created weekly sampling assignments from ArcGIS Workforce's web-based map. The sample collection teams used ArcGIS Workforce's mobile app to view assignments, get driving directions, and open ArcGIS Field Maps mobile app. ArcGIS Field Maps allowed teams to view and interact with City of Atlanta's sewerage system. A custom URL was created

and linked to a form in ArcGIS Survey123. The custom URL was configured to communicate attributes of the sampled manhole (e.g., manhole ID, pipe size etc.) to ArcGIS Survey123 and avoid teams having to copy and paste data. Collection date and time, team members ID, and geographic location information were automatically populated when the form in ArcGIS Survey123 opened. Teams then filled out a few questions, took photos of sampling site, and scanned the barcodes on the bottles or collection bags. The barcode number was then populated in the form. The teams submitted the forms in the field using cellular data and then continued to the next assigned sampling site. Later, the teams entered the date and time the bottles were delivered to the lab at Emory University for testing. ArcGIS Survey123 created a point feature in a feature layer in ArcGIS Online. These feature layers were analyzed in ArcGIS Pro and exported in multiple data formats. Results from the lab were then populated in ArcGIS Online. ArcGIS Dashboards used the feature layer to display the data. Widgets in ArcGIS Dashboards were configured to sort the data by date, council district, and neighborhood planning unit (NPU). The integration between multiple apps and the seamless transfer of data is the reason why Esri Field Operations Apps were chosen. The required training was minimal due to the intuitive work flow of the apps.

B Supplementary Figures

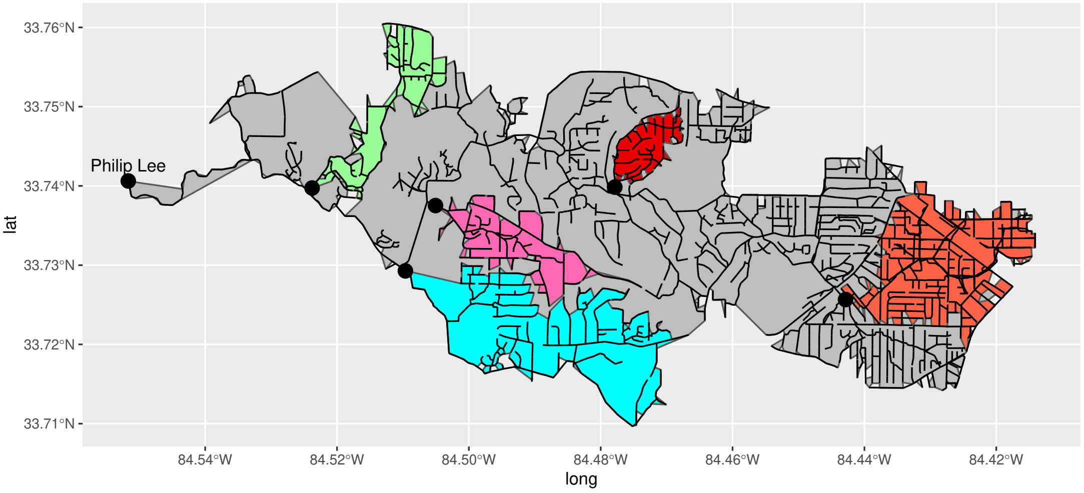

Supplementary Figure 1. Illustration of nested sampling design for the Phillip Lee sampling cluster, including one influent line site and five independent community sites nested within the catchment area of the Phillip Lee influent line site. The gray polygon represents the catchment area of the Phillip Lee influent line site. The five polygons with different colors represent catchment areas of specific community manhole sites.

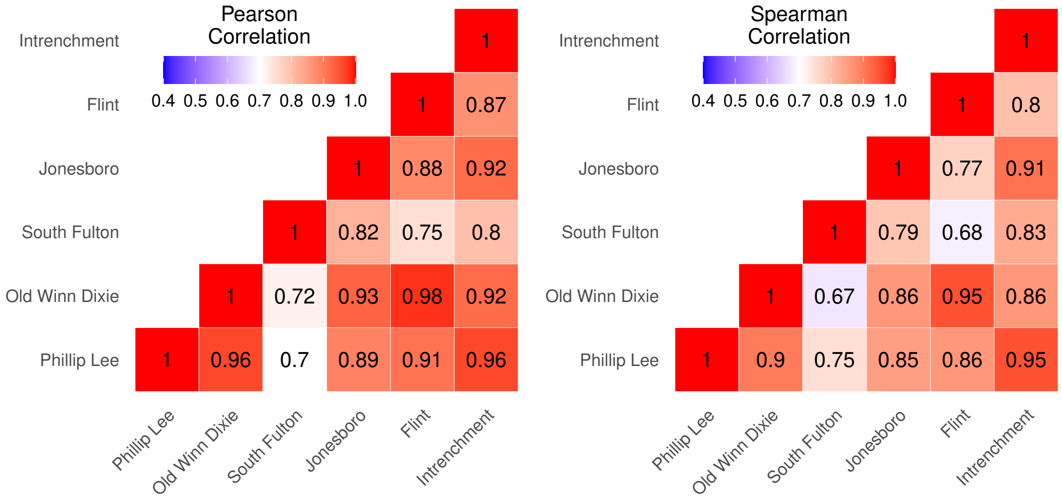

Supplementary Figure 2. Correlations between SARS-CoV-2 RNA concentrations in the wastewater samples from six influent line sites.

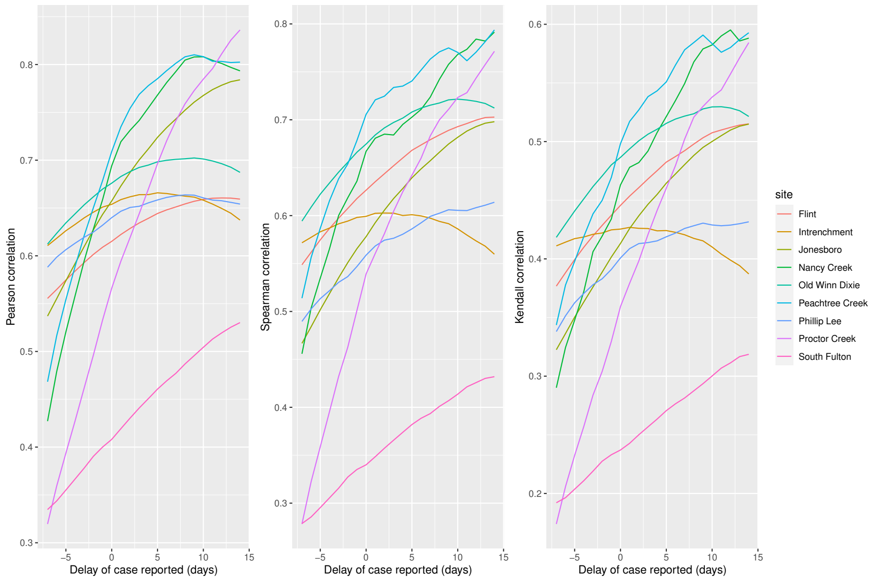

Supplementary Figure 3. Correlations between SARS-CoV-2 RNA concentrations in the wastewater samples and delayed reported case numbers in Fulton County by influent line site.

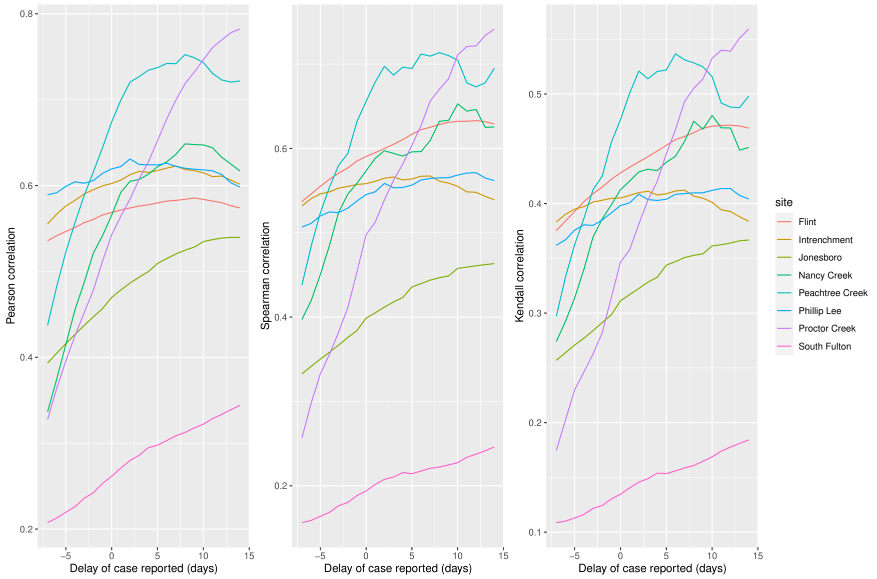

Supplementary Figure 4. Correlations between SARS-CoV-2 RNA concentrations in the wastewater samples and delayed reported case numbers in the influent line catchment area by influent line site.

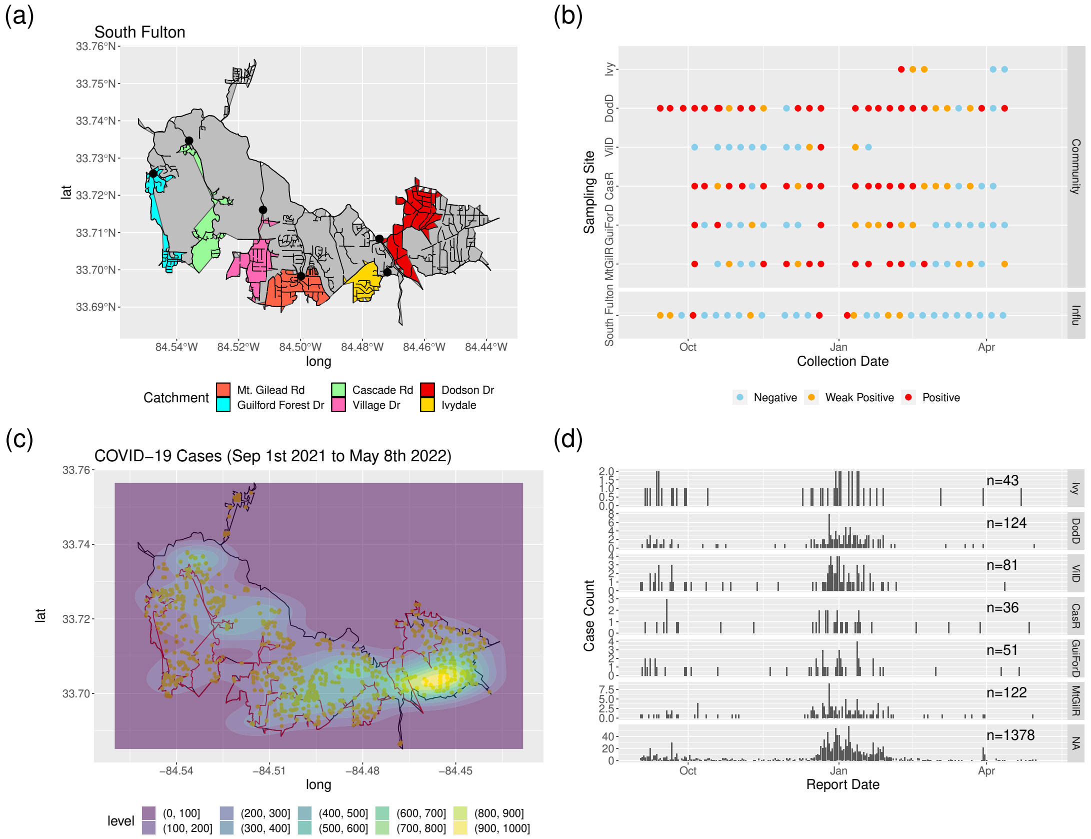

Supplementary Figure 5. SARS-CoV-2 wastewater surveillance results for community sites and reported COVID-19 case numbers in the catchment area for South Fulton sampling cluster between September 2021–May 2022. Subfigure (a) shows the catchment areas of each community site nested within the overall catchment of the influent line site (in gray). The black lines represent the sewer network lines. Subfigure (b) shows the weekly wastewater surveillance results (RT-PCR detection of SARS-CoV-2 RNA). Subfigure (c) shows the heatmap of reported COVID-19 cases between September 1st 2021–May 8th 2022 within the influent catchment area. Subfigure (d) shows the epidemic curves of COVID-19 within each community site catchment area and NA represents all the cases in the catchment area of the influent line site that are not in the catchment area of a specific community site. MtGilR, GuiForD, CasR, VilD, DodD, and Ivy represent Mt. Gilead Rd, Guilford Forest Dr, Cascade Rd, Village Dr, Dodson Dr, and Ivydale respectively.

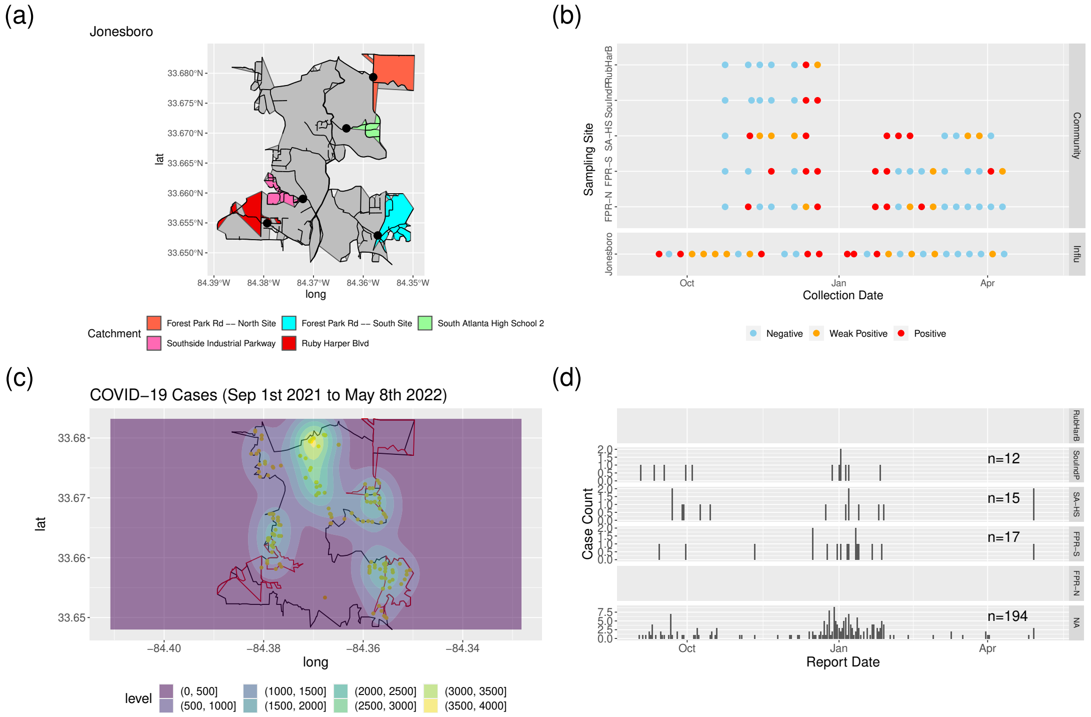

Supplementary Figure 6. SARS-CoV-2 wastewater surveillance results for community sites and reported COVID-19 case numbers in the catchment area for Jonesboro sampling cluster between September 2021–May 2022. Subfigure (a) shows the catchment areas of each community site nested within the overall catchment of the influent line site (in gray). The black lines represent the sewer network lines. Subfigure (b) shows the weekly wastewater surveillance results (RT-PCR detection of SARS-CoV-2 RNA). Subfigure (c) shows the heatmap of reported COVID-19 cases between September 1st 2021–May 8th 2022 within the influent catchment area. Subfigure (d) shows the epidemic curves of COVID-19 within each community site catchment area and NA represents all the cases in the catchment area of the influent line site that are not in the catchment area of a specific community site. FPR-N, FPR-S, SA-HS, SouIndP, and RubHarB represent Forest Park Rd – North Site, Forest Park Rd – South Site, South Atlanta High School, Southside Industrial Parkway, and Ruby Harper Blvd respectively.

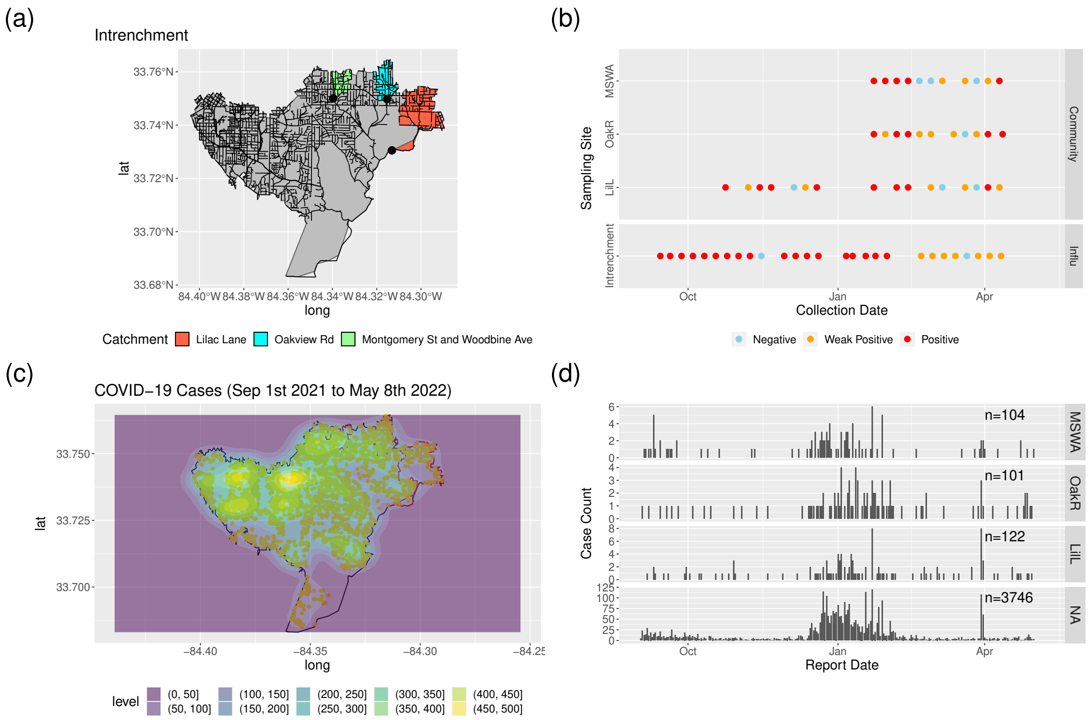

Supplementary Figure 7. SARS-CoV-2 wastewater surveillance results for community sites and reported COVID-19 case numbers in the catchment area for Intrenchment sampling cluster between September 2021–May 2022. Subfigure (a) shows the catchment areas of each community site nested within the overall catchment of the influent line site (in gray). The black lines represent the sewer network lines. Subfigure (b) shows the weekly wastewater surveillance results (RT-PCR detection of SARS-CoV-2 RNA). Subfigure (c) shows the heatmap of reported COVID-19 cases between September 1st 2021–May 8th 2022 within the influent catchment area. Subfigure (d) shows the epidemic curves of COVID-19 within each community site catchment area and NA represents all the cases in the catchment area of the influent line site that are not in the catchment area of a specific community site. LilL, OakR, and MSWA represent Lilac Lane, Oakview Rd, and Montgomery St and Woodbine Ave respectively.

C Supplementary Tables

Supplementary Table 1. Descriptive information for influent lines. WRC represents Water Reclamation Center.

WRC	Influent Line	Cover Combined Sewer Area	Cover Industrial Area	# of Manholes Covered	Estimated Catchment Population	ZIP Codes Covered
Utoy Creek	Phillip Lee	No	No	3640	37,552	30311, 30314, 30331, 30310
	Old Winn Dixie	No	No	13	NA	30331
	South Fulton	No	No	2489	39,377	30311, 30331, 30310
South River	Jonesboro	No	Yes	770	5427	30354, 30315
	Flint	No	Yes	2959	29,519	30354, 30315, 30312, 30304, 30303, 30349, 30313, 30310
	Intrenchment Creek	Yes	Yes	7091	79,502	30032, 30315, 30316, 30307, 30312, 30303, 30317, 30313, 30310
R.M. Clayton	Nancy Creek	No	No	3602	41,698	30318, 30319, 30326, 30327, 30305, 30342
	Proctor Creek	Yes	Yes	5923	51,433	30314, 30318, 30303, 30308, 30313, 30310
	Peachtree Creek	Yes	Yes	11,279	178,077	30309, 30318, 30307, 30319, 30312, 30303, 30326, 30327, 30308, 30317, 30306, 30305, 30324, 30342

Supplementary Table 2. Descriptive information for community sites. *The catchment size are calculated as the number of manholes covered.

Name	Type	Manhole ID	Catchment Size*	Associated with Influent Line
1295 West Apartments	Apartment Complex	23150234301	12	Phillip Lee
Cascade Glen Apartments	Apartment Complex	13940319801	1	South Fulton
Columbia Tower at MLK Village	Apartment Complex	23360349501	13	Intrenchment Creek
Country Oaks	Apartment Complex	Unable to identify	NA	Phillip Lee
Fairburn & Gordon II Apartments	Apartment Complex	Unable to identify	NA	NA
Fairway Gardens Apartments	Apartment Complex	Unable to identify	NA	NA
Heritage Station Apartments	Apartment Complex	Unable to identify	NA	NA
Life at Greenbriar Apartments	Apartment Complex	Unable to identify	NA	NA
Pavillion Place Apartments	Apartment Complex	23230107301	50	NA
Peyton Village Apartments	Apartment Complex	Unable to identify	NA	NA
Venetian Hills Apartments	Apartment Complex	23140107401	85	South Fulton
Veranda at Auburn Point	Apartment Complex	23360357501	7	Peachtree Creek
Metropolitan Gardens Condominiums	Apartment Complex	Unable to identify	NA	NA
815 Old Flat Shoals Road	Apartment Complex	23360472211	2	NA
Atlanta Industrial Parkway	Community	13980407601	7494	NA
Benjamin E Mays	Community	13960301201	45	Phillip Lee
Benjamin E Mays High School	Community	13950110501	187	Phillip Lee

Continued on next page

Continued from previous page

Name	Type	Manhole ID	Catchment Size*	Associated with Influent Line
Butler Way NW	Community	23080119001	127	NA
Cambridge Dr & Hogan Rd SW	Community	13930103001	132	NA
Cascade Falls	Community	13950413001	24	Phillip Lee
Cascade Rd	Community	13850100701	235	South Fulton
Chatham Ave	Community	23150132801	733	Phillip Lee
Dodson Dr	Community	23040112201	256	South Fulton
Eloise St SE & Mercer St SE	Community	23350230103	221	Intrenchment Creek
Engelwood Ave SE & Boulevard SE	Community	23350400404	3	Intrenchment Creek
Fair Street SW & Agnes Jones Place	Community	23160442201	105	Proctor Creek
Fairburn Rd	Community	13950100901	221	South Fulton
Forest Park Rd – North Site	Community	23330214201	35	Jonesboro
Forest Park Rd – South Site	Community	23320205701	87	Jonesboro
Guilford Forest Dr	Community	13850103501	222	South Fulton
Ivydale	Community	23040107701	114	South Fulton
Larchwood	Community	23060408001	62	Phillip Lee
Lilac Lane	Community	23550100401	354	Intrenchment Creek
Montgomer St and Woodbine Ave	Community	23460337501	187	Intrenchment Creek
Mt. Gilead Rd	Community	13940200401	259	South Fulton
Oakview Rd	Community	23560310401	217	Intrenchment Creek

Continued on next page

Continued from previous page

Name	Type	Manhole ID	Catchment Size*	Associated with Influent Line
Parsons St SW & Lawshe St SW	Community	23260351103	455	Proctor Creek
Peeples St SW & Cunningham Place	Community	23160455101	108	Proctor Creek
Peyton Woods Trail	Community	23060310701	98	Phillip Lee
Plainville Trail	Community	13860413001	182	Phillip Lee
Pryor St & Richardson St	Community	23260408201	765	Intrenchment Creek
Rockwell St & Coleman St	Community	23250100000	NA	NA
Ruby Harper Blvd	Community	23320107001	44	Jonesboro
Sandy Creek	Community	13970109201	1409	NA
Simon St	Community	23320107101	54	Jonesboro
South Atlanta High School 1	Community	23250320901	1	Flint
South Atlanta High School 2	Community	23330200801	32	Jonesboro
Southside Industrial Parkway	Community	23330305601	60	Jonesboro
Spink St NW	Community	23080102101	60	NA
Victoria Place	Community	23250135301	6	Phillip Lee
Village Dr	Community	13950301801	209	South Fulton
Walmart	Community	13950102901	661	Phillip Lee
Gateway Capitol View	Community	23150422001	1	Flint
Belmonte Hills Townhomes	Community	23150137701	25	Phillip Lee
Cascade Commons	Community	Unable to identify	NA	NA

Continued on next page

Continued from previous page

Name	Type	Manhole ID	Catchment Size*	Associated with Influent Line
Oakland Place Townhomes	Community	23150432601	1	Phillip Lee
Wildwood Townhomes	Community	Unable to identify	NA	NA
